# The *xantha* Marker Trait Is Associated with Altered Tetrapyrrole Biosynthesis and Deregulated Transcription of PhANGs in Rice

**DOI:** 10.3389/fpls.2017.00901

**Published:** 2017-05-31

**Authors:** Rui-Qing Li, Meng Jiang, Yan-Hua Liu, Yun-Chao Zheng, Jian-Zhong Huang, Jian-Min Wu, Qing-Yao Shu

**Affiliations:** ^1^National Key Laboratory of Rice Biology, Institute of Crop Sciences, Zhejiang UniversityHangzhou, China; ^2^Hubei Collaborative Innovation Center for Grain IndustryJingzhou, China; ^3^Department of Chemistry, Zhejiang UniversityHangzhou, China; ^4^Institute of Nuclear Agricultural Sciences, Zhejiang UniversityHangzhou, China

**Keywords:** chlorophyll, tetrapyrrole, photosynthesis, retrograde signaling, PhANGs, hybrid rice, seed purity

## Abstract

The *xantha* marker trait, which is controlled by a down-regulating epi-mutation of *OsGUN4*, has been applied to the production of hybrid rice. However, the molecular basis for the ability of *xantha* mutants to attain high photosynthetic capacity even with decreased chlorophyll contents has not been characterized. In the present study, we observed that the total chlorophyll content of the *xantha* mutant was only 27.2% of that of the wild-type (WT) plants. However, the *xantha* mutant still accumulated 59.9% of the WT δ-aminolevulinic acid content, 72.8% of the WT Mg-protoporphyrin IX content, and 63.0% of the WT protochlorophyllide *a* content. Additionally, the protoporphyrin IX and heme contents in the mutant increased to 155.0 and 160.0%, respectively, of the WT levels. A search for homologs resulted in the identification of 124 rice genes involved in tetrapyrrole biosynthesis and photosynthesis. With the exception of *OsGUN4*, *OsHO-1*, and *OsHO-2*, the expression levels of the genes involved in tetrapyrrole biosynthesis were significantly higher in the *xantha* mutant than in the WT plants, as were all 72 photosynthesis-associated nuclear genes. In contrast, there were no differences between the *xantha* mutant and WT plants regarding the expression of all 22 photosynthesis-associated chloroplast genes. Furthermore, the abundance of ^1^O_2_ and the expression levels of ^1^O_2_-related genes were lower in the *xantha* mutant than in the WT plants, indicating ^1^O_2_-mediated retrograde signaling was repressed in the mutant plants. These results suggested that the abundance of protoporphyrin IX used for chlorophyll synthesis decreased in the mutant, which ultimately decreased the amount of chlorophyll in the *xantha* mutant. Additionally, the up-regulated expression of photosynthesis-associated nuclear genes enabled the mutant to attain a high photosynthetic capacity. Our findings confirm that OsGUN4 plays an important role in tetrapyrrole biosynthesis and photosynthesis in rice. GUN4, chlorophyll synthesis pathways, and photosynthetic activities are highly conserved in plants and hence, novel traits (e.g., *xantha* marker trait) may be generated in other cereal crops by modifying the *GUN4* gene.

## Introduction

Hybrid rice is important for rice production in China and other countries (reviewed by Zhang, 2016). The leaf color marker trait has been proposed as a genetic marker for male-sterile lines, which can simplify the differentiation between bred-true and off-type (unwanted) plants, thus achieving high varietal purity during rice production (Shu et al., 1996). To date, male-sterile lines with marker traits, such as *chlorina* (Dong et al., 1995), *xantha* (Zhou et al., 2006a), green-revertible albino seedlings (Wu et al., 2003, 2011), and purple plants (Mou et al., 1995), have been developed and used to produce hybrid rice.

We previously generated a *xantha* marker trait through γ-irradiation of Longtefu B (LTB), which is a rice cytoplasmic male-sterile maintainer line (Zhou et al., 2006a). Plants of the *xantha* mutant line, named Huangyu B (HYB), have yellow leaves, and can be visually distinguished from normal green rice plants. Although HYB contains lower levels of chlorophyll *a* (Chl *a*), chlorophyll *b* (Chl *b*), and carotenoids (Car) than LTB, it retains a high photosynthetic rate comparable to that of wild-type (WT) plants at high-light intensities (Zhou et al., 2006b; Wu et al., 2007). Thus, HYB differs from most chlorophyll-deficient mutants, which typically exhibit relatively low photosynthetic activities (Liu et al., 2007, 2016; Yoo et al., 2009; Tian et al., 2013; Wang et al., 2014; Zhang et al., 2015; Yang et al., 2016). Therefore, the *xantha* marker trait is very unique and valuable for breeding hybrid rice varieties. It has been introduced into several cytoplasmic male-sterile lines and thermosensitive genic male-sterile lines, including Huangyu A (Zhou et al., 2006a), Jiazhe91A (Li et al., 2010), and LFS (Fu Haowei, personal communication).

**FIGURE 1:**
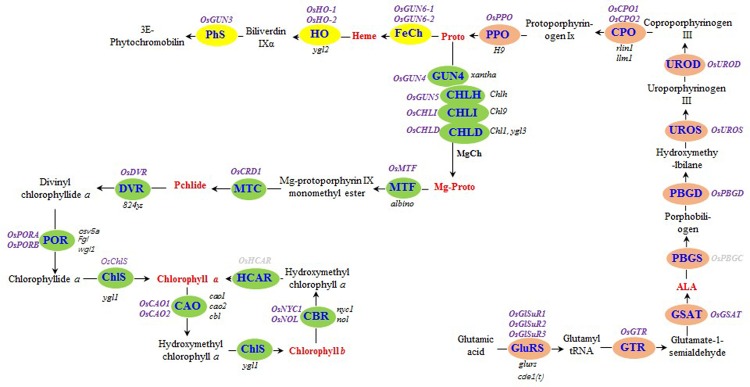
Tetrapyrrole biosynthesis pathway in rice. The enzymes of the tetrapyrrole biosynthesis pathway are indicated by blue text. The steps before the tetrapyrrole branch are presented in orange. The Mg and Fe branches are indicated in green and yellow, respectively. The rice genes encoding tetrapyrrole biosynthesis-associated enzymes are listed in purple (bold), while the rice mutants are listed in black (italics). The seven tetrapyrrole intermediates detected in this study are presented in red (bold). Rice genes encoding hydroxymethyl chlorophyll *a* reductase and porphobilinogen synthase were not detected, and are indicated in gray (bold). GluRS, glutamyl-tRNA synthetase; GTR, glutamyl-tRNA reductase; GSAT, glutamate-1-semialdehyde aminotransferase; ALA, δ-aminolevulinic acid; PBGS, porphobilinogen synthase; PBGD, porphobilinogen deaminase; UROS, uroporphyrinogen III synthase; UROD, uroporphyrinogen III decarboxylase; CPO, coproporphyrinogen III oxidase; PPO, protoporphyrinogen IX oxidase; Proto, protoporphyrin IX; MgCh, protoporphyrin IX Mg-chelatase; ChlH, Mg-chelatase H subunit; ChlI, Mg-chelatase I subunit; ChlD, Mg-chelatase D subunit; Mg-Proto, Mg-protoporphyrin IX; MTF, Mg-protoporphyrin IX methyltransferase; MTC, monomethyl ester oxidative cyclase; DVR, divinyl chlorophyllide *a* 8-vinyl reductase; POR: NADPH, protochlorophyllide oxidoreductase; ChlS, chlorophyll synthase; CAO, chlorophyllide *a* oxygenase; CBR, chlorophyll *b* reductase; HCAR, hydroxymethyl chlorophyll *a* reductase; FeCh, protoporphyrin IX Fe-chelatase; HO, heme oxygenase; PhS, phytochromobilin synthase; Pchlide, protochlorophyllide *a*.

Chlorophyll biosynthesis involves many enzymes (Beale, 2005; Tanaka and Tanaka, 2006) that catalyze the conversion of the first committed precursor, δ-aminolevulinic acid (ALA), to the final products in higher plants, namely Chl *a* and Chl *b* (**Figure 1**). Mutations to any of these genes may reduce or even eliminate chlorophyll biosynthesis, leading to leaf color changes (Liu et al., 2007, 2016; Yoo et al., 2009; Tian et al., 2013; Wang et al., 2014; Zhang et al., 2015; Yang et al., 2016). Through gene mapping and additional studies, the mutant gene responsible for the *xantha* marker trait of HYB was identified as a mutated *OsGUN4* (*genomes uncoupled 4*) (Chi et al., 2010; Li et al., 2014).

In higher plants, more than 95% of chloroplast proteins are encoded by nuclear genes, and chlorophyll biosynthesis is closely associated with the expression of photosynthesis-associated nuclear genes (PhANGs) (Sawchuk et al., 2008; Pogson et al., 2008). The coordinated expression between chloroplast genes and PhANGs is achieved through plastid-to-nucleus retrograde signaling pathways (reviewed by Chan et al., 2016; Larkin, 2016). The GUN4 protein is involved in chlorophyll biosynthesis and retrograde signaling in *Arabidopsis thaliana* (Larkin et al., 2003; Strand et al., 2003; Peter and Grimm, 2009; Adhikari et al., 2011; Richter et al., 2016), *Chlamydomonas reinhardtii* (Formighieri et al., 2012; Brzezowski et al., 2014; Tabrizi et al., 2015), *Synechocystis* sp. (Sobotka et al., 2008; Chen et al., 2015) and rice (Zhou et al., 2012). It has been documented that GUN4 stimulates Mg chelatase activity in rice (Zhou et al., 2012) and other angiosperms (Richter et al., 2016). In HYB, mutations to *OsGUN4* result in a significant decrease in chlorophyll content (Li et al., 2014), which also demonstrated the role of OsGUN4 in chlorophyll biosynthesis.

The *xantha* marker also is associated with a very unique trait, in which decreased chlorophyll content is coupled with a high photosynthetic capacity (Zhou et al., 2006b; Wu et al., 2007). To elucidate the biological mechanisms underlying the *xantha* marker trait, we examined the differences in tetrapyrrole synthesis, photosynthesis, and retrograde signaling molecules between the *xantha* mutant and its WT parent. We revealed that altered tetrapyrrole biosynthesis and deregulated transcription of PhANGs might be responsible for the *xantha* marker trait.

## Materials and Methods

### Plant Growth Conditions

The *xantha* mutant line HYB and its WT parental line LTB were previously developed by our group (Zhou et al., 2006a). Seedlings of the two lines were planted in plastic barrels filled with soil from a rice paddy at the Zhejiang University experimental farm. The seedlings were grown in a growth chamber under a 16-h light (1,000 μmol photons m^-2^ s^-1^; 30°C):8-h dark (26°C) photoperiod.

### Analysis of Chlorophyll and Tetrapyrrole Intermediates

We maintained 32-day-old seedlings in 1× Murashige and Skoog liquid medium for 3 days. Leaf tissue was collected at midday to analyze chlorophyll and tetrapyrrole intermediate contents. The Chl and Car contents were determined using 0.5 g fresh leaf tissue according to a published method (Peter and Grimm, 2009). The Chl *a*, Chl *b*, and Car concentrations were calculated according to the method described by Lichtenthaler (1987).

The ALA content was measured using a slightly modified version of the procedure described by Czarnecki et al. (2012). Briefly, 0.5 g fresh leaf tissue was homogenized in liquid nitrogen and suspended in 500 μL acetate buffer (57 μL acetic acid, 100 μM sodium acetate, 200 μL acetoacetic ester, pH 4.6, and double-distilled H_2_O up to 500 μL). The sample was boiled in a water bath for 15 min and then cooled to room temperature. We added 1 mL acetyl ethyl ester to the cooled solution, which was then incubated at room temperature for 5 min. After a 5-min centrifugation at 16,000 ×*g*, the supernatant was transferred to a new tube, and mixed with 200 μL reagent (1 g 4-dimethylaminobenzaldehyde, 5 mL perchloric acid, 5 mL H_2_O, and acetic acid up to 50 mL). The optical density of the solution was measured at 553 nm, and the ALA concentration was determined using a standard curve generated with commercial ALA (Sigma–Aldrich Inc., Shanghai, China).

The protoporphyrin IX (Proto), Mg-protoporphyrin IX (Mg-Proto), and protochlorophyllide *a* (Pchlide) contents were determined using a commercial enzyme-linked immunosorbent assay kit (Jingmei Tech., China) (Engvall and Perlmann, 1971). Briefly, 0.5 g fresh leaf tissue ground in liquid nitrogen was suspended in 1 mL cold alkaline acetone containing 0.1 N NH_4_OH (9:1; v/v) and then centrifuged (16,000 ×*g* for 5 min). We extracted Proto (Papenbrock et al., 1999), Mg-Proto (Papenbrock et al., 1999), and Pchlide (Koski and Smith, 1948) from the supernatant. The pellets were suspended in 1 mL acetone/HCl/DMSO (10:0.5:2, v/v/v) and centrifuged (16,000 ×*g* for 5 min) three times. The heme content of the supernatant was determined as previously described by Peter and Grimm (2009).

### Identification of Genes Involved in Tetrapyrrole Biosynthesis and Photosynthesis

The rice genes encoding enzymes involved in tetrapyrrole biosynthesis were identified either through BLAST searches of *A. thaliana* protein sequences in the National Center for Biotechnology Information database or using gene names (Supplementary Table S1) in the Rice Annotation Project database^1^. We identified 30 genes (**Figure 1** and Supplementary Table S1).

Plant photosynthesis takes place in Photosystems I and II (PSI and PSII) and in the cytochrome (cyt) *b_6_f* complex (Supplementary Figure S1A). PSI consists of the PsaA–L, PsaN, and PsaO subunits, and the light-harvesting chlorophyll-binding proteins Lhca1–4 (Supplementary Figure S1A). PSII contains major intrinsic proteins (Supplementary Figure S1A), such as PsbA (D1), PsbB (CP47), PsbC (CP43), and PsbD (D2), as well as the PsaE and PsbF polypeptides, cyt *b_559_*, and the O_2_ evolution activity unit (PsaO, PsaP, and PsbQ). The PsbS subunit and other polypeptides (PsbH–N, PsbR, PsbT, and PsbW–Z) are also included. The electron transport chain of rice chloroplasts includes (Mn)_4_, P680, Phe, *Q*_A_, and *Q*_B_ from PS II; PQH2 and PC from cyt *b_6_f*; and P700, *A*_0_, *A*_1_, *F*_x_, *F*_A_, *F*_B_, *F*_d_, and FNR from PS I. The Rubisco (Rbc) enzyme consists of five small subunits (RbcS) and one large subunit (RbcL) (Supplementary Figure S1B). We identified 90 photosynthesis-associated genes based on BLAST searches (Supplementary Table S1).

There are two types of RNA polymerase in plastids, i.e., nuclear-encoded plastid RNA polymerase (NEP) and plastid-encoded plastid polymerase (PEP). The sole NEP encoding gene (*OsRopTp*), two plastid genes that encode the subunits of PEP (*OsrpoA and OsrpoB*), two nuclear genes that encode PEP associated proteins (*OsSIG5 and OsSIG6*) were also identified (Supplementary Table S1).

### Quantitative Real-Time Polymerase Chain Reaction Analysis

Total RNA was extracted from fresh leaf tissue using the Spin Plant RNA Mini Kit (Qiagen, Germany). The leaf tissue was collected at midday from seedlings that had been grown in liquid medium for 3 days after being transferred from soil. The cDNA was reverse transcribed using 1 μg total RNA, an oligo-dT_18_ primer, and the GoScript^TM^ Reverse Transcription System (Promega). The quantitative real-time polymerase chain reaction (RT-q PCR) was conducted using a SYBR Green GoTaq^®^ qPCR Master Mix containing the ROX reference dye (Promega, Beijing, China). The rice *ACTIN* gene (*Os03g0718100*) was used as an internal control, and the relative expression levels were calculated according to the 2^-ΔΔCt^ method (Livak and Schmittgen, 2001). Gene details and qRT-PCR primer sequences are provided in Supplementary Table S1.

### Determination of Singlet Oxygen Content

The singlet oxygen (^1^O_2_) content was determined using the singlet oxygen sensor green fluorescent probe (Hideg et al., 2002). Leaves sampled at midday from seedlings grown in liquid medium were stored at -70°C until used. Frozen rice leaves were ground in liquid nitrogen and suspended with 1 mL pre-chilled lysis buffer (GENMED Scientifics Inc., United States). The mixture was briefly vortexed and immediately centrifuged at 4°C (5,000 ×*g* for 10 min). The supernatant was transferred to a new pre-chilled 1.5-mL tube and mixed with a solution consisting of 10 μmol singlet oxygen sensor green probe (GENMED Scientifics Inc.) and 0.02% methanol. The fluorescence spectra were analyzed at excitation and emission wavelengths of 485 and 520 nm, respectively, using a 359S fluorescence spectrophotometer (Lengguang Tech., China).

### Statistical Analysis

All measurements were completed using six biological replicates, each consisting of pooled tissue from three seedlings. Statistical analyses were conducted using the *F*-tests and Student’s *t*-test.

## Results

### The *xantha* Mutation Altered the Biosynthesis of Tetrapyrrole

**FIGURE 2:**
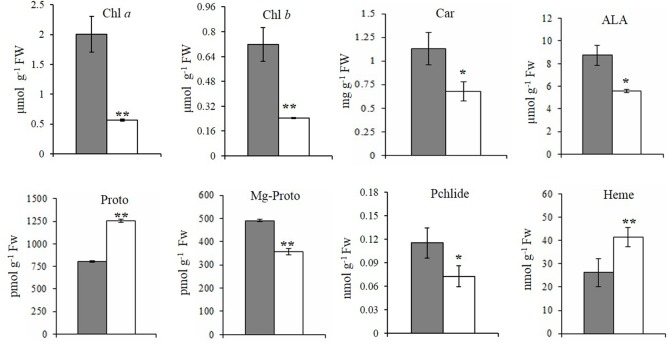
Steady-state levels of tetrapyrrole intermediates in rice. The steady-state levels of intermediates in wild-type plants (gray bar) and *xantha* mutants (open bar) were analyzed using six biological replicates. Error bars represent the standard error. ^∗^, ^∗∗^, and ^∗∗∗^ represent significant differences between wild-type and *xantha* mutant values at *P* < 0.05, *P* < 0.01, and *P* < 0.001, respectively.

Similar to previous observations, field-grown *xantha* mutant plants exhibited yellow leaf color phenotypes (Supplementary Figures S2) and had significantly lower Chl *a*, Chl *b*, and Car contents than the WT controls (**Figures 2A–C**). Consequently, the Chl *a*/*b* and Car/Chl ratios were 1.8- and 11.3-fold higher for the *xantha* mutant than for the WT plants, respectively.

To clarify how chlorophyll contents decreased, the contents of five tetrapyrrole intermediates were also analyzed. First, the ALA and Proto contents exhibited the opposite trends in the *xantha* mutant, with ALA levels decreasing by 40.1% (**Figure 2D**) and Proto levels increasing by 55.0% (**Figure 2E**) relative to the WT levels. In the Mg-Proto branch, the contents of Mg-Proto (**Figure 2F**) and Pchlide (**Figure 2G**) decreased by 27.2 and 37.0%, respectively. In the Fe-Proto branch, the *xantha* mutant accumulated 1.6-fold more heme than the WT controls (**Figure 2H**). These results indicated that the *xantha* mutation altered tetrapyrrole biosynthesis, with more intermediates diverted to the Fe-Proto branch.

**FIGURE 3:**
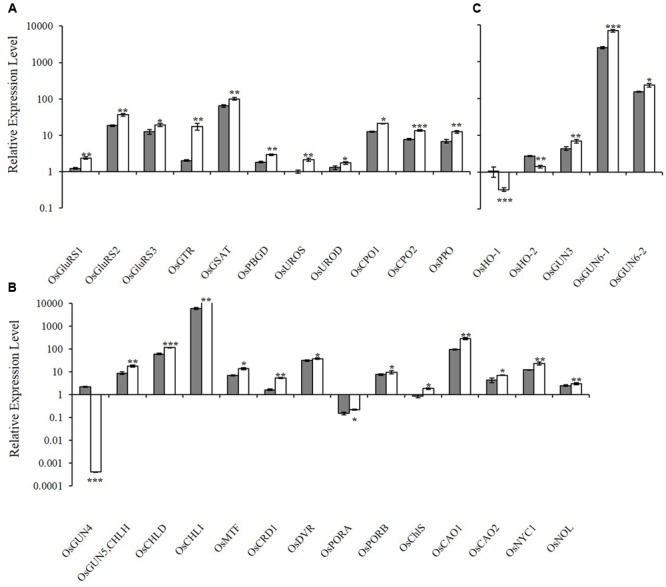
Relative expression levels of tetrapyrrole biosynthesis-associated genes in rice. **(A)** Expression levels of genes encoding enzymes for the shared steps of the tetrapyrrole biosynthesis pathway. **(B)** Expression levels of genes from the Mg branch. **(C)** Expression levels of the genes from the Fe branch. The transcript levels in wild-type plants (gray bar) and *xantha* mutants (open bar) were first normalized to the internal control gene *OsACTIN* and reported relative to the *OsUROS* level in wild-type plants (assigned a value of 1). Error bars represent the standard error. ^∗^, ^∗∗^, and ^∗∗∗^ represent significant differences between wild-type and *xantha* mutant values at *P* < 0.05, *P* < 0.01, and *P* < 0.001, respectively.

To determine how the *xantha* mutation altered tetrapyrrole biosynthesis, we investigated the effects of the *xantha* mutation on the transcription of 30 rice genes involved in different tetrapyrrole biosynthesis steps (**Figure 1**). Consistent with the results of our previous studies, the abundance of *OsGUN4* transcripts in the *xantha* mutant was only 0.02% of that of the WT plants (**Figure 3**). Surprisingly, the abundance of all transcripts except for those of *OsHO-1* and *OsHO-2* was significantly greater in the *xantha* mutant than in the WT controls (**Figure 3**). Additionally, the transcripts of genes involved in shared steps of the tetrapyrrole biosynthesis pathway were 1.3–8.6-fold more abundant in the *xantha* mutant than in the WT plants (**Figure 3A**), while the transcripts of genes in the Mg-Proto branch were 1.2–3.2-fold more abundant in the *xantha* mutant (**Figure 3B**). In the Fe-Proto branch, the *OsGUN3*, *OsGUN6-1*, and *OsGUN6-2* expression levels were up-regulated in the *xantha* mutant, with 1.5–2.9-fold higher transcript levels than in the WT plants. In contrast, *OsHO-1* and *OsHO-2* expression was down-regulated (**Figure 3C**).

### The *xantha* Mutation Up-regulated the Transcription of PhANGs, But Had No Effect on Chloroplast Genes

**FIGURE 4:**
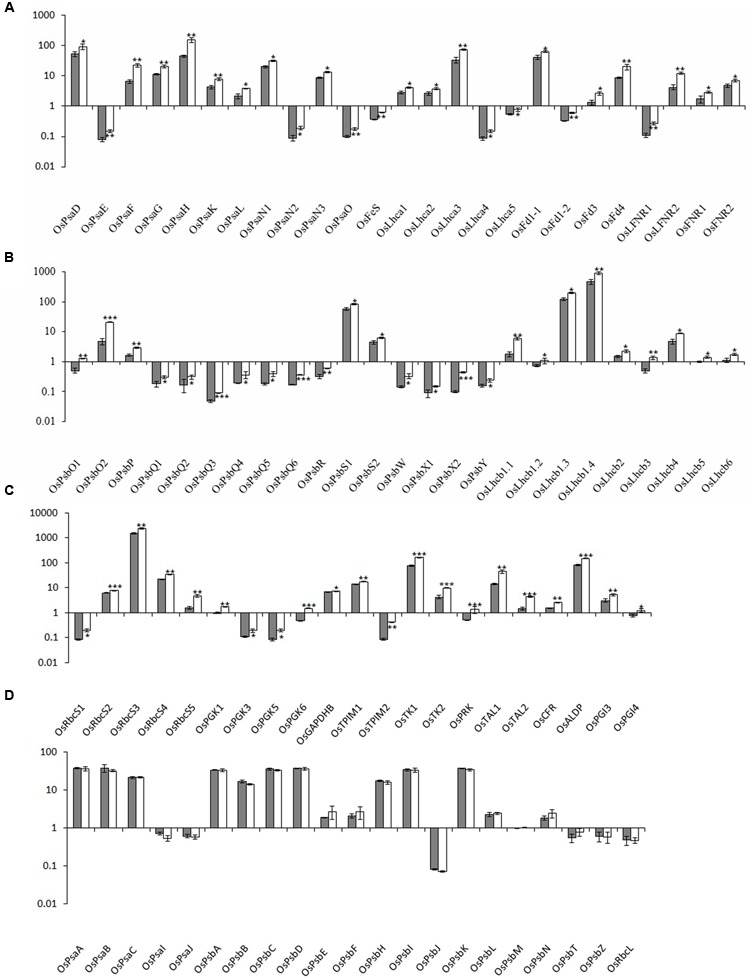
Relative expression levels of photosynthesis-associated nuclear genes in rice. The transcript levels of PhANGs for PSI **(A)**, PSII **(B)**, and the CO_2_ fixation system **(C)** in wild-type plants (gray bar) and *xantha* mutants (open bar) were analyzed using six biological replicates. **(D)** The transcript levels of photosynthesis-associated chloroplast genes (PhACGs) for PSI, PSII, and the CO_2_ fixation system in wild-type plants (gray bar) and *xantha* mutants (open bar). The transcript levels were first normalized to the internal control gene *OsACTIN* and reported relative to the *OsLhcb5* level for nuclear genes and to *OsPsbM* for plastid genes in wild-type plants (assigned a value of 1), respectively. Error bars represent the standard error. ^∗^, ^∗∗^, and ^∗∗∗^ represent significant differences between wild-type and *xantha* mutant values at *P* < 0.05, *P* < 0.01, and *P* < 0.001, respectively.

To characterize the biological basis underlying the high photosynthetic efficiency of the *xantha* mutant, the transcription of photosynthesis-associated genes (i.e., PhANGs and chloroplast genes) was investigated. Overall, the expression levels of all PhANGs were significantly higher in the *xantha* mutant than in the WT, while there were no differences in the chloroplast gene expression levels (**Figure 4**).

We analyzed five chloroplast genes (*OsPsaA–C*, *OsPsaI*, and *OsPsaJ*) and 25 PhANGs in PSI (Supplementary Table S1 and Figure S1A). The PhANG expression levels were 1.5–3.5-fold higher in the *xantha* mutant than in the WT plants, whereas there were no significant differences in the chloroplast gene expression levels between the mutant and WT plants (**Figures 4A,D**). Additionally, the expression levels of five *Lhca* genes (*OsLhca1–5*) and the genes encoding electron transport chain components were up-regulated by 1.4–2.2-fold and 1.4–2.9-fold, respectively (**Figure 4A**).

We identified 41 PSII genes (16 chloroplast genes and 25 PhANGs) (Supplementary Table S1 and Figure S1A). The transcription level changes of PSII genes were similar to those of PSI genes. The transcripts for the genes encoding subunit and Lhcb proteins were 1.4–4.4-fold and 1.4–3.4-fold higher in the *xantha* mutant than in the WT plants, respectively (**Figure 4B**). However, there was no difference between the *xantha* mutant and WT plants regarding the expression of chloroplast genes encoding PSII subunits (**Figure 4D**).

One chloroplast gene (*OsRbcL*) and 22 nuclear genes (five *OsRbcSs* and 17 enzyme-encoding genes) were identified for the CO_2_ fixation system. Of these genes, only three (*OsGAPD*, *OsTK1*, and *OsCFR*) were previously identified in rice (Supplementary Table S1 and Figure S1B). The transcript levels for the *RbcS* genes and the 17 enzyme-encoding genes were 1.3–3.0-fold and 1.1–4.8-fold higher in the *xantha* mutant than in the WT plants, respectively (**Figure 4C**). However, similar to the other chloroplast genes, there was no difference in the transcription of *RbcL* between the *xantha* mutant and WT plants (**Figure 4D**). Thus, compared with the WT levels, the expression levels of all of the PhANGs were up-regulated in the *xantha* mutant, while there were no differences in the expression of the chloroplast genes.

The transcription of NEP/PEP and PEP associated genes were also investigated. No significant differences of transcript level were observed for the two plastid PEP genes, while the three nuclear genes had higher levels of transcript in the *xantha* mutant than in the WT plants (Supplementary Figure S3).

### The *xantha* Mutation Decreased ^1^O_2_ Production and Down-regulated ^1^O_2_-Dependent Retrograde Signaling

**FIGURE 5:**
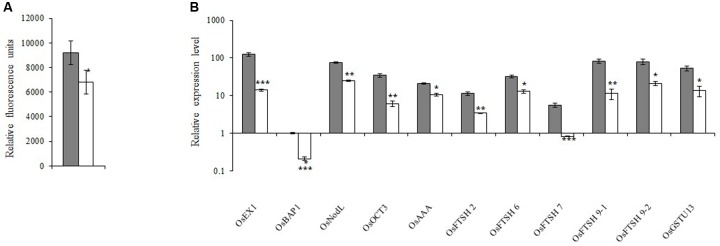
^1^O_2_-inducible retrograde signaling pathway in rice. **(A)** ^1^O_2_ concentrations in *xantha* mutant and wild-type plants. The accumulation of ^1^O_2_ in six biological replicates was quantified based on singlet oxygen sensor green fluorescence. Error bars represent the standard error. ^∗^Represents a significant difference between wild-type and mutant values at *P* < 0.05. **(B)** Expression levels of genes involved in the ^1^O_2_-inducible retrograde signaling pathway in wild-type plants (gray bar) and *xantha* mutants (blank bar) were first normalized to the *OsActin* internal control and were reported relative to the wild-type *OsBAP* expression level (assigned a value of 1). Error bars represent the standard error. ^∗^, ^∗∗^, and ^∗∗∗^ represent significant differences between wild-type and *xantha* mutant values at *P* < 0.05, *P* < 0.01, and *P* < 0.001, respectively.

Because ^1^O_2_ is believed to be important for retrograde signaling, we measured the ^1^O_2_ concentration. We observed that the ^1^O_2_ concentration in the *xantha* mutant was 38.0% lower than that of the WT seedlings (**Figure 5A**). Decreases in the ^1^O_2_ level should affect the expression of the genes in the ^1^O_2_-dependent signaling pathway. Thus, 11 such genes were identified and their transcription levels were examined. Consistent with the lower ^1^O_2_ content, the transcript abundance for these genes in the *xantha* mutant was only 11.0–50.0% of that of the WT plants (**Figure 5B**).

## Discussion

The function of GUN4 during the regulation of chlorophyll synthesis and in plastid-to-nucleus retrograde signaling has been studied in *A. thaliana*, *C. reinhardtii* and *Synechocystis* sp., but little is known regarding the GUN4 homologs in rice and other higher plants. Based on our previous observation that an epi-mutation of *OsGUN4* results in the *xantha* marker trait, we investigated the effects of OsGUN4 on tetrapyrrole biosynthesis and the regulation of genes involved in photosynthetic activities by comparing the *xantha* mutant and its WT parent. Our results may be useful for elucidating the molecular mechanism responsible for the *xantha* phenotype in rice plants. They may also provide novel insights into the function of GUN4 in plants beyond the model plant species *A*. *thaliana.*

### Effect of the *xantha* Mutation on Tetrapyrrole Synthesis

The GUN4 protein was first identified as a regulator of chlorophyll biosynthesis (Larkin et al., 2003) in *A. thaliana*, mainly at the posttranslational level (Peter and Grimm, 2009). Many *GUN4* mutants have been identified in *A. thaliana* (Larkin et al., 2003; Adhikari et al., 2011) and *C*. *reinhardtii* (Brzezowski et al., 2014). Knockout mutants [e.g., *gun4-2* (Larkin et al., 2003) and *line 026911* (Peter and Grimm, 2009)] exhibit an albino phenotype and do not accumulate chlorophyll under dark–light growth conditions. However, other *A. thaliana* mutants, such as *gun4-1* (L88F amino acid change; Larkin et al., 2003) and *line 011461* (truncated GUN4), contain chlorophyll, but only at approximately 40% (Mochizuki et al., 2001) and 50% (Peter and Grimm, 2009) of the WT level, respectively. The differences in chlorophyll content between *gun4* mutants and WT *A. thaliana* plants diminish with decreasing light intensity (Peter and Grimm, 2009). There are also fewer differences in *C*. *reinhardtii* under dark vs. continuous light conditions (Brzezowski et al., 2014). Although the epi-mutation almost eliminates *OsGUN4* expression at the transcript and protein levels (Li et al., 2014), rice *xantha* mutant plants still accumulate chlorophyll, albeit at 37.2% of the WT level (**Figures 2A,B**). This suggests that OsGUN4 and GUN4 have similar effects on chlorophyll biosynthesis.

*In vitro* activity studies have already demonstrated that OsGUN4 promotes magnesium chelatase activity in rice (Zhou et al., 2012). However, when its C-terminal domain was absent or phosphorylated (Zhou et al., 2012; Richter et al., 2016), it no more activates magnesium chelatase. This not only demonstrated the essential role of its C-terminal domain, but also explained different *gun4* mutations could have different effects on chlorophyll biosynthesis.

The observation that the *xantha* mutant accumulated significantly less ALA and more Proto than the WT plants (**Figure 1**) is consistent with the results for the *gun4* mutants of *A. thaliana* (Peter and Grimm, 2009; Czarnecki et al., 2012) and *C*. *reinhardtii* (Brzezowski et al., 2014). However, the *xantha* mutant accumulated significantly more heme than the WT control plants (**Figure 2H**), which is in contrast to what was observed for the mutant *A. thaliana line 011461* (Peter and Grimm, 2009). In *C*. *reinhardtii gun4* mutants, the reported changes in the heme level were not consistent. For example, Formighieri et al. (2012) concluded the per-cell heme level did not increase, while Brzezowski et al. (2014) reported that significantly more heme accumulated in the *gun4* mutant than in the WT plants under continuous light conditions, but no differences were observed in plants grown in darkness.

In the Mg-Proto branch, the Mg-Proto and Pchlide contents were significantly lower in the *xantha* mutant than in the WT plants (**Figures 2F,G**). The abundance of all tetrapyrrole intermediates increased in transgenic tobacco plants overexpressing the *A. thaliana GUN4* gene (Peter and Grimm, 2009). Meanwhile, the changes to Mg-Proto and Pchlide levels were only analyzed for one *A. thaliana gun4* knockout mutant grown on medium supplemented with 250 μM ALA. The mutant plants accumulated more Mg-Proto and less Pchlide than the WT controls after a 24-h incubation in darkness. However, after a 24-h incubation under dim light, the trend was reversed (Peter and Grimm, 2009).

The chlorophyll deficiency in the *xantha* mutant may be due to the decreased abundance of Mg-Proto and Pchlide. The *OsGUN4* epi-mutation resulted in an extremely low level of OsGUN4 in the *xantha* mutant (Li et al., 2014). Hence the mutation would greatly reduce its stimulatory effect on Mg-chelatase activity, and consequently lead to a decrease in the amount of Proto being diverted to the Mg-Proto branch and an increase of Proto accumulation (**Figures 1**, **2**). The decreased accumulation of ALA in the *xantha* mutant was also consistent with the increased heme level, because heme reportedly inhibits ALA synthesis (Apitz et al., 2016). Other metabolites, e.g., heme catabolites may also play a role in the regulation of ALA synthesis.

### Mutated *OsGUN4* Deregulates the Transcription of Nuclear Genes Involved in Tetrapyrrole Synthesis and Photosynthesis

In the tetrapyrrole biosynthesis pathway, there are 11 genes encoding proteins/enzymes for shared steps, 14 for the Mg-Proto branch and five for the Fe-Proto branch (**Figure 1**). The transcription of all tetrapyrrole biosynthesis genes except for *OsHO-1* and *OsHO-2* was significantly up-regulated in the *xantha* mutant (**Figure 3**). A similar observation was reported for a *C. reinhardtii gun4* mutant, although the *HO* genes were not examined (Brzezowski et al., 2014). These results suggest that the effects of *GUN4* on tetrapyrrole biosynthesis are highly conserved. This phenomenon has yet to be examined in *gun4* mutants of *A. thaliana* and other higher plants.

The expression of PhANGs is closely associated with chlorophyll biosynthesis through plastid-to-nucleus retrograde signaling mediated by *genomes uncoupled* genes (Chi et al., 2010; Chan et al., 2016). Similar to the tetrapyrrole biosynthesis genes, the transcription of all 122 PhANGs was significantly higher in the *xantha* mutant than in the WT plants, implying that the expression of PhANGs was deregulated (**Figure 4**). A similar observation was reported for a *C. reinhardtii gun4* mutant (Brzezowski et al., 2014). In sharp contrast, there were no significant differences between the *xantha* mutant and WT plants regarding the transcript abundance for the photosynthesis-associated chloroplast genes (PhACGs) (**Figure 4**), suggesting that PhACGs and PhANGs are independently regulated.

There are two types of RNA polymerase in chloroplast but only PEP transcribes PSI and PSII genes (Kremnev and Strand, 2014; Borner et al., 2015). The two PEP encoding plastid genes (i.e., *OsrpoA* and *OsrpoB*) had similar transcript levels in the *xantha* mutant and in the WT plants (Supplementary Figure S3). These results are not only consistent with the present observation that the *xantha* mutant and WT plants had similar transcriptional levels of PhACGs (**Figure 4**), but also support previous conclusions (Borner et al., 2015). The core PEP enzyme requires a sigma factor for promoter recognition and initial of transcription; *OsSig5* and *OsSig6* are the two genes potentially encoding potential plastid sigma factors of RNA polymerase in rice (Kubota et al., 2007), their transcript levels were significantly greater in the *xantha* mutant than in the WT plants (Supplementary Figure S3), which suggests that it is the core units of PEP, not the sigma factor, that determine the transcription of PhACGs.

### Role of OsGUN4 in Plastid-to-Nucleus Retrograde Signaling

When the *GUN4* gene was first identified in *A. thaliana*, its function in retrograde signaling was confirmed (Larkin et al., 2003). However, there is still a lack of consensus about its exact role in this process. Two contrasting hypotheses have been proposed based on *in vitro* and *in vivo* studies involving *A. thaliana* and *C. reinhardtii.* Most studies suggested that GUN4 ‘shields’ Proto and Mg-Proto from collisions with O_2_, thereby decreasing the production of reactive oxygen species (ROS) (e.g., ^1^O_2_) (Larkin et al., 2003; Sobotka et al., 2008), which are important retrograde signaling molecules. Brzezowski et al. (2014) proposed that *C. reinhardtii* GUN4 functions as a potential sensor of excess Proto resulting from perturbations in the tetrapyrrole biosynthesis pathway, and as a mediator of the Proto-triggered ^1^O_2_-dependent retrograde signaling pathway. In contrast, Tabrizi et al. (2016) suggested that the light-dependent generation of ^1^O_2_ by GUN4–Proto may represent the first step of retrograde signaling from the chloroplast to the nucleus. In this proposal, GUN4 is involved in increasing rather than decreasing ^1^O_2_ contents.

The *xantha* mutant accumulated less Mg-Proto and more Proto than the WT plants (**Figures 2B,C**). Because OsGUN4 is almost undetectable in the *xantha* mutant (Li et al., 2014), if OsGUN4 is mainly responsible for shielding Proto and MgProto as suggested by Brzezowski et al. (2014), then an increase in ROS and ^1^O_2_ production would be expected. However, if Tabrizi et al. (2016) are correct, a decrease in the ^1^O_2_ content of the *xantha* mutant would be expected because of a lack of or a very limited amount of GUN4-MgProto. Therefore, the significantly lower ^1^O_2_ abundance in the *xantha* mutant than in the WT plants (**Figure 5A**) supports the GUN4 function proposed by Tabrizi et al. (2016). The effects of decreased ^1^O_2_ abundance in the *xantha* mutant are also reflected by the down-regulated transcription of genes associated with the ^1^O_2_-dependant signaling pathway (**Figure 5B**). Thus, these results help to explain the deregulation of PhANGs due to chlorophyll deficiencies in the *xantha* mutant.

A new model of tetrapyrrole-dependent plastid-to-nucleus signaling was recently proposed by Larkin (2016). It suggests that heme accumulates in active chloroplasts, and that the accumulation or export of heme to the cytosol or nucleus activates a mechanism that induces the expression of PhANGs. The observed elevated heme content (**Figure 2H**) and increased transcription of PhANGs (**Figures 4A,B**) in the *xantha* mutant are consistent with this model.

### High Photosynthetic Capacity in *gun4* Mutants

Although its chlorophyll content is only 37.2% of that of WT plants, the *xantha* mutant retains a high photosynthetic capacity (Zhou et al., 2006a; Wu et al., 2007). The effects of *gun4* mutations on photosynthetic activities were briefly discussed for *C. reinhardtii* (Formighieri et al., 2012) and in more detail for *A. thaliana* (Daloso et al., 2014).

An earlier study (Zhou et al., 2006a) involving field-grown plants revealed that the *xantha* mutant had a higher net photosynthetic rate (*P*_N_) than WT plants, possibly because of its lower non-photochemical quenching (*q*_N_) value. Additionally, the maximal photochemical efficiency (*F*_V_/*F*_M_), excitation energy capture efficiency of PSII reaction centers (F_V_′/F_M_′), photochemical quenching (*q*_P_), effective PSII quantum yield (Φ_PS2_), and non-cyclic electron transport rate (ETR) were all higher in the *xantha* mutant than in the WT line. Wu et al. (2007) conducted a diurnal study on the photosynthetic activities of the *xantha* mutant under field conditions at high temperatures and irradiance. Photo-inhibition was observed for the WT line above 1,043 μmol photons m^-2^ s^-1^, but not for the mutant (the highest light intensity during the day was 1,235 μmol photons m^-2^ s^-1^). During the photo-inhibitory period for the WT line, the mutant exhibited higher *P*_N_, *q*_N_, *F*_V_/*F*_M_, Φ_PS2_, and ETR values. These observations can be partially explained by chlorophyll changes in the mutant. For example, the lower Chl *b* content in the mutant is likely associated with a lower capacity for photon capture. Thus, PSII would be more stable under high-light intensities, resulting in an enhanced Φ_PS2_ (Wu et al., 2007). The present study revealed that the transcription of PhANGs was significantly higher in the *xantha* mutant than in the WT plants (**Figures 4A,B**), which may have contributed to the high photosynthetic capacity of the mutant. For example, the up-regulated expression of *LHCB* genes may optimize the transfer of light energy from light-harvesting complex II to the reaction center of PSII, thereby contributing to the ETR, as suggested by Daloso et al. (2014) for *A. thaliana*.

Previous studies involving *A. thaliana* and *C. reinhardtii gun4* mutants produced in consistent results regarding the effect of GUN4 on photosynthesis. In *A. thaliana*, Daloso et al. (2014) reported higher Φ_PS2_ and *q*_P_ values for a *gun4* mutant. This suggests the mutant transfers the excess absorbed light energy more efficiently than the WT plants despite a decrease in chlorophyll content. The mutant also likely has a greater capacity to dissipate excess absorbed energy. Although the *F*_V_/*F*_M_ values were similar, the Φ_PS2_ value was higher for the WT *C. reinhardtii* (0.46) than for the *gun4* knockout mutant (0.32) (Formighieri et al., 2012). The differences between the *gun4* mutant and WT plants are often dependent on environmental conditions. For example, they become significant under high-light intensities (in rice) or after exposure to continuous light stress (in *A. thaliana*), which is probably because of the photo-protective function of GUN4 (Formighieri et al., 2012; Brzezowski et al., 2014). Therefore, the inconsistencies in the reported results are not unexpected.

### Future Studies on GUN4 in Crop Plants

Since the initial discovery of *A. thaliana* GUN4 in 2003 (Larkin et al., 2003), follow-up studies have been conducted only for *C. reinhardtii*, *A. thaliana*, and *Synechocystis* sp. There were no reports describing GUN4 from other plant species until we revealed that an epi-allele of *OsGUN4* is responsible for the *xantha* marker trait (Li et al., 2014). This previous study remains the only one involving a GUN4 from a crop plant. Because GUN4 is important for regulating chlorophyll and tetrapyrrole syntheses, as well as photosynthetic activities (Larkin, 2016), additional studies on GUN4 in crop plants, including rice, may help to more comprehensively characterize GUN4 functions. They may also provide new insights into crop productivity and environmental adaptability. Furthermore, the genetic manipulation of the *GUN4* gene may generate new *GUN4* alleles useful for improving the ability of crops to adapt to particular environments.

Although the GUN4 proteins of crop plants likely function similarly to the GUN4 of *A. thaliana* and *C. reinhardtii*, they may also have additional or different roles. For example, we observed that the *xantha* mutant had a significantly increased heme level (**Figure 2H**), which was not observed in *A. thaliana* and *C. reinhardtii gun4* mutants (Peter and Grimm, 2009; Formighieri et al., 2012; Brzezowski et al., 2014). Most of the studies of *A. thaliana* GUN4 were performed under artificial conditions, with relatively low light intensities. Therefore, the photo-protective role of GUN4 needs to be examined in crop plants grown under field conditions with natural sunlight.

In summary, our present study extended the research on GUN4 to a crop plant, and elucidated the molecular basis of the first agronomic trait regulated by an epi-allele of the *GUN4* gene. We observed that a mutated *OsGUN4* altered tetrapyrrole biosynthesis and deregulated the transcription of PhANGs. Our data confirm that OsGUN4 is important for tetrapyrrole biosynthesis and photosynthetic activities in rice plants.

## Author Contributions

Q-YS and J-MW conceived the study. R-QL and MJ conducted the RT-q PCR experiments. R-QL and Y-CZ completed the bioinformatic analyses. R-QL, Y-HL, and MJ analyzed the tetrapyrrole intermediates and ^1^O_2_. R-QL, Q-YS, and J-ZH analyzed the data. R-QL and J-ZH drafted the manuscript, and Q-YS prepared the final version. All authors reviewed and approved the final manuscript.

## Conflict of Interest Statement

The authors declare that the research was conducted in the absence of any commercial or financial relationships that could be construed as a potential conflict of interest.

## Footnotes

1 http://rapdb.dna.affrc.go.jp/
