# Supplementary material for: The xantha Marker Trait Is Associated with Altered Tetrapyrrole Biosynthesis and Deregulated Transcription of PhANGs in Rice
Source: Front Plant Sci. 2017 May 31;8:901. doi: 10.3389/fpls.2017.00901 (PMC5449477; doi:10.3389/fpls.2017.00901)
Supplement: Supplementary file 1 [file Table_1.PDF]

Supplementary Table 1|Rice genes involved in tetrapyrrole biosynthesis, photosynthesis, plastid-to-nucleus retrograde signaling and in plastid gene transcription

Gene Symbol[a]	Gene code	Encoded/putative prtotein[b]	Forward (F) and reverse (R) primers for RT-q PCR (5'→3')
1. Genes involved in tetrapyrrole biosynthesis pathway			
OsGluRS1	Os01g0271200	Glutamyl-tRNA synthetase	F: ACCACTTGCCATCCTCTGTG<br>R: ACCAAGGTGAAGTGTGGGTG
OsGluRS2	Os02g0121000	Glutamyl-tRNA synthetase [1]	F: CCAAACACATTACGGCAGGC<br>R: CTGTCCCACAGAAGTAGCCC
OsGluRS3	Os10g0369000		F: GCAATGCTGTGTGAGGGGTA<br>R: GCGCATCAACGAATGGACAA
OsGTR	Os10g0502400	Glutamyl-tRNA reductase	F: TGCTATGGGTGGTGTTCGAC<br>R: CTTCTTGGTGAGGGCGTCTT
OsGSAT	Os08g0532200	Glutamate semialdehyde aminotransferase	F: AATGTGTTGGCTGAGATGGTC<br>R: TGCATGGCCATGGTAACAAC
OsPBGD	Os02g0168800	Porphobilinogen deaminase	F: CTGAAGGCTGCACACTCAGA<br>R: TTTCATCGAGTGGACGGCAA
OsUROS	Os03g0186100	Uroporphyrinogen-III synthase	F: CAGATAGGCTTTCTGCTGTCCT<br>R: TACTTCGTCAAAAACTCTTTCA
OsUROD	Os03g0337600	Uroporphyrinogen-III decarboxylase	F: ATTGCTCTGCGAGCGACATC<br>R: TCGGGCTTGAAAACCTTCCA
OsCPO1	Os04g0610800	Coproporphyrinogen III oxidase[2-3]	F: CACCCCAAGAACCCATTTGC<br>R: CACCAGGAGCATCTTTCGGT
OsCPO2	Os12g0270100		F: TATCAGGTTCTCCAGGCCCA<br>R: ACTCTGTCAGACTCCTTCCGA
OsPPO	Os10g0473800	Protoporphyrinogen IX oxidase[4]	F: TCGTTCGCTCTGTCCGCTTT<br>R: GTGAGAGAAAACGGTACGGG
OsGUN4	Os11g0267000	Genome uncoupled 4 [5]	F: TTACCGGCAGGCCGACGAGA<br>R: TGCCCAGGAGCTGTGTCCCT
OsGUN5	Os03g0323200	Subunit H of Mg-chelatase[6]	F: CTATACATTCGCCACACT<br>R: TATCACACAACTCCCAAG
OsCHLD	Os03g0811100	Subunit D of Mg-chelatase[7-8]	F: GAGGTCCTCGACTCCACCAAC<br>R: CCACGAGCCATCACTGTCT
OsCHLI	Os03g0563300	Subunit I of Mg-chelatase[8-9]	F: AGTAACCTTGGTGCTGTG<br>R: AATCCATCAACATTCAACTCTG
OsMTF	Os06g0132400	Mg protoporphyrin IX methytransferase[10]	F: GACGTGCTGATCCACTACCC<br>R: CTGATGAGCACCCGCTTCT
OsMTC	Os01g0279100	Monomethyl ester oxidative cyclase	F: CTCAACCAGGAGGAGTTCGAC<br>R: GAGAGGCCCCTCCATCTTGTC
OsDVR	Os03g0351200	Divinyl chlorophyllide a 8-vinyl reductase[11]	F: CAAGGGGAGATGCTGTTCCG<br>R: CCATGCCTTCCCTGATCACC
OsPORA	Os04g0678700	Protochlorophyllide oxidoreductase A[12]	F: TGCTCTCTCTGTCCCCAAGA<br>R: TCTGGCTCACGCTAAGGAAC

OsPORB	Os10g0496900	Protochlorophyllide oxidoreductase B[13-14]	F: ACCACCACCTCCTTCCTCC<br>R: CAAGAACGCCGAGTCCTTCA
OsChlS	Os05g0349700	Chlorophyll a synthase[15]	F: ACAAAGAGACGAAGGCCAATG<br>R: ATGTCGTTCTCTTGCTTGGCG
OsCAO1	Os10g0567400	Chlorophyllide a oxygenase[16]	F: TTGCCAAGGGTTGGAGTGTT<br>R: GAGACATCCGGTAGAGCAGC
OsCAO2	Os10g0567100	Chlorophyllide a oxygenase[17]	F: AAACCACTGTGTCTTTGCTGC<br>R: ATATCGACGACCAATGCCCA
OsNYC1	Os01g0227100	Chlorophyll b reductase[18]	F: TCGGTTGCTACGCAAGTAGG<br>R: AAGGAACTCCCGAGCAAGTG
OsNOL	Os03g0654600	Chlorophyll b reductase[19]	F: CGGAGGCTGTCCCTGGT<br>R: CCTTTCGTGGAACCGGTGAT
OsHO-1	Os06g0603000	Heme oxgenase 1[20]	F: CAAGGAAGGGGAGAAGGAGC<br>R: GACGAGGAAGCGGAGGTAG
OsHO-2	Os03g0395000	Heme oxgenase 2	F: GGGACCTAGCAGCCCTAAC<br>R: GGGGTTCCGGAGGCG
OsGUN3	Os01g0949400	Genome uncoupled 3	F: TGCGCCAACGTTTTTACAACT<br>R: GGTGGAGCCAGGAAAGATCC
OsGUN6-1	Os09g0297000	Genome uncoupled 6	F: GGGGACGAGTTCCTGCATTA<br>R: TACGCCGCTAGCAGTTCTTC
OsGUN6-2	Os05g0361200		F: GGGTGTAAAGAGCCTCCTGG<br>R: GAACCCTTCCCCAGTTCTCG
2. Photosynthesis-associated genes			
2.1 Photosystem I			
OsPsaA	OrsajCp025	PSI subunit PsaA[21]	F: AGGCCCAGACAAAATGAGCA<br>R: CGATTAATGGGTGGCTCCGA
OsPsaB	OrsajCp024	PSI subunit PsaB[22]	F: AGCCAAAGGTGTCCGTTCAT<br>R: GGTATGGGCGTGGATGTTCT
OsPsaC	OrsajCp089	PSI subunit PsaC[23-24]	F: AAGATAGAGCCATGCTGCGG<br>R: CGCCAAGAACCGAAGATTGT
OsPsaD	Os08g0560900	PSI subunit PsaD[23-24]	F: CGCAGGTGGAGGAGTTCTAC<br>R: CCCGTGGGCATCTCGAAC
OsPsaE	Os07g0435300	PSI subunit PsaE	F: CTCGAGGTTCCCGCCG<br>R: GGACTCCCTCCTCAGGATCT
OsPsaF	Os03g0778100	PSI subunit PsaF[25]	F: TCCAGTCGTCGCTCAAGAAGTA<br>R: TTCGTCTTCTCGATGGTGGCG
OsPsaG	Os09g0481200	PSI subunit PsaG	F: TCAAGAGAGCACCACCACTC<br>R: GTGGCGGCCATGGTT
OsPsaH	Os05g0560000	PSI subunit PsaH[26]	F: GAGGACATCGGCAACACCAC<br>R: CCGAGCAGCAGGAACTTGAG
OsPsaI	OrsajCp037	PSI subunit PsaI	F: ACCCTCTATTTTCGTGCCTTT<br>R: TGCACATAAAGAAATAAAGAAG
OsPsaJ	OrsajCp048	PSI subunit PsaJ	F: ATGCGGGATATAAAAACATA<br>R: AATGACAAAGCATCTGGAAA

OsPsaK	Os07g0148900	PSI subunit PsaK	F: CTCCTAGGTCCATGGTGACG<br>R: ACCATGATAACGTTGGTGGCT
OsPsaL	Os12g0420400	PSI subunit PsaL	F: TGATGAAGAGCAGCTTGGTGT<br>R: TCAGACTGGATGGCCTTGAC
OsPsaN1	Os03g0731100	PSI subunit PsaN	F: ATCAGGAGGCCATTCACAGC<br>R: AGAAGGCCCCTCCTGCTAAT
OsPsaN2	Os05g0242400		F: GCTCCTGGTGAACTGTCGTC<br>R: GCTGACGAGGTTGTTGTTCAC
OsPsaN3	Os12g0189400		F: GACGAGTACCTCGAGAAGAGC<br>R: CCGGTGAAGTTGTAGGGGAAC
OsPsaO	Os04g0414700	PSI subunit PsaO	F: CCTTCTCCACCGGCTTCAC<br>R: CTAAGCCAGTCCTGCGGAAA
OsFeS	Os05g0157300	4Fe-4S ferredoxin	F: GGACTGGGTCAAGCTCATCT<br>R: ACCTTCATTCACTGCACCCA
OsLhca1	Os06g0320500	Light-harvesting complex I	F: GTCGTCGAACAGGAGCCG<br>R: GGTCGAACCCGAAGTCACC
OsLhca2	Os07g0577600	Chlorophyll a-b binding protein[27]	F: CGACAGGCCAATCTGGTTCC<br>R: TCCGATCCGAGACCCAGG
OsLhca3	Os02g0197600	Chlorophyll a/b-binding protein type III	F: AGGCTCTTCTCTCTGGGAGG<br>R: GAACCACAGCTGCCTGTCA
OsLhca4	Os08g0435900	LHC I type IV chlorophyll binding protein	F: CCTTCCTAGGGCAATCCTCC<br>R: CCGTTGAGGTAGGTGGGAGA
OsLhca5	Os02g0764500	Lhca 5 protein	F: CTCTCCCCAAAGCCGGTTAC<br>R: CGAGCTCGTGGAGACTGAAA
OsFd1-1	Os08g0104600	Ferredoxin I, chloroplast precursor	F: GGGATCGACCTGCCTTACTC<br>R: GGAAGCTCTGGTCGGACTG
OsFd1-2	Os04g0412200		F: CCGTCTACACGGTGAAGCTC<br>R: GCGTCCAGGATGTAGGTGTC
OsFd3	Os03g0835900	Ferredoxin III, chloroplast precursor	F: AGCTTCTTCAGCCAAGGCAT<br>R: TGCCCTTCTGGTGTGATGAG
OsFd4	Os05g0443500	Ferredoxin VI, chloroplast precursor	F: TGTCAGGCCTAAGGATCTCCA<br>R: TTTCAGCGGCCTCGAGAATG
OsLFNR1	Os02g0103800	Ferredoxin-$NADP^+$ oxidoreductase [28]	F: TCCTGAACACGAGGATCACC<br>R: CTTGCCGTTCTTGTCGATGC
OsLFNR2	Os06g0107700		F: CTACAGAGAGGGGCAGTCCA<br>R: ACGCAGAGTGAAACGGTCTT
OsFNR1	Os03g0784700	Ferredoxin-NADP reductase[29]	F: CCTTACTGGGAGGGGCAAAG<br>R: AGACGGACATTATGTGGTGC
OsFNR2	Os07g0147900		F: TTCCCCAAATACTGTCCGGC<br>R: CGACGAACACACAAACTGGC
2.2 Photosystem II			
OsPsbA	OrsajCp002	Photosystem II protein D1[30]	F: GAGCAGCTAGGTCTAGAGGGA<br>R: GTTGATAGCCAAGGTCGCGT
OsPsbB	OrsajCp053	Photosystem II protein CP47	F: TGATCCGGGTCGATTGCTTT

			R: TCAGAGGGATCAAAAACCGCT
OsPsbC	OrsajCp009	Photosystem II protein CP43[31]	F: TCGTGACCAAGAAACCACCG<br>R: CATTGCTCCGGCCCAGAATA
OsPsbD	OrsajCp008	Photosystem II protein D2[32]	F: GACTGGTTACGAAGGGACCG<br>R: TGTCCCTGTAAACCAACCTCC
OsPsbE	OrsajCp045	Photosystem II protein E	F: GTCTGGAAGCACGGGAGAAC<br>R: ACGGAATTCCTTGTCGGCTT
OsPsbF	OrsajCp044	Photosystem II protein F	F: GACCATAGATCGAACCTAT<br>R: CGTTGGATGAACTGCATTGC
OsPsbH	OrsajCp056	Photosystem II protein H	F: CTGGACCAAGACAAACTCGC<br>R: CAACCCGGAGCTACTTTCCC
OsPsbI	OrsajCp006	Photosystem II protein I	F: ACTCTCAAACTTTTTGT<br>R: CGTCACGCCCAGGATTAC
OsPsbJ	OrsajCp042	Photosystem II protein J[33]	F: ACCCAATCCAGAATATGAACCGT<br>R: TGGCTGATAGGTACTGTAACCG
OsPsbK	OrsajCp005	Photosystem II protein K[34]	F: TTTCTTCGCCAAATTGCCCG<br>R:CAGCTTGCCAAACAAAGGCT
OsPsbL	OrsajCp043	Photosystem II protein L[33]	F: TGACACAATCAAACCCGAATGA<br>R: ACAAAAATGAGTAATAAACCCCAGT
OsPsbM	OrsajCp013	Photosystem II protein M	F: TGCAAGTCCTCCCCTTGAAT<br>R: GCAGTAGCAATAAATGCGAG
OsPsbN	OrsajCp055	Photosystem II protein N	F: AACAGCAACTTTAGTCGCCA<br>R: TGTTGAGAGGGTTGCCCAAA
OsPsbO1	Os01g0501800	Photosystem II protein O[35]	F: CCACCTCAGCTCTGCT<br>R: TGAATCTCGTCGAAGGTAAG
OsPsbO2	Os04g0414700		F: ATCACCTGCATGACGTTTC<br>R: ATCCACCCGATCAGCC
OsPsbP	Os07g0141400	Photosystem II protein P	F: GAGTTCATCGCCTACAGC<br>R: GAACTCGCGCTCCTTG
OsPsbQ1	Os02g0578400	Photosystem II protein Q	F: ACGAGGTCGTACGTCAG<br>R: GTGCGCATCCCCTCC
OsPsbQ2	Os02g0631100		F: TCTGCCGGATCAAGAACT<br>R: GTCGTCCATGCCAATCAC
OsPsbQ3	Os04g0522800		F: ATTTGTCGGATCAACAACTG<br>R: CACGAGTCCTCATCATCAAT
OsPsbQ4	Os04g0523000		F: ATGGCATTGCAGCTCG<br>R: CTCCGTTGGCCGTTTC
OsPsbQ5	Os05g0313100		F: ATGGCATTGCAGCTCG<br>R: CTCCGTTGGCCGTTTC
OsPsbQ6	Os07g0105600		F: ATGGCCACTTATCTCCAATC<br>R: CCACGAGGAGGAGCAG
OsPsbR	Os07g0147500		F: AACCATCTGCATCTCCATTAC<br>R: ACTATGATCAAAGATGGCCTTG
OsPsbS1	Os01g0869800	22-kDa Photosystem II	F: CTGTTCGGCAGGTCCAAGAC

		protein[36]	R: AGGATTCCCTTCCCGGTGAT
OsPsbS2	Os04g0690800	22-kDa Photosystem II protein[37]	F: TTCCCCCAAGGTGGACAAGT<br>R: CGGCCTCGTAGATGGGGATG
OsPsbT	OrsajCp054	Photosystem II protein T	F: TGGAAGCATTGGTTTATACGTTCC<br>R:GGAACCTTAGGTGGTTCTCGG
OsPsbW	Os01g0773700	Photosystem II protein W	F: ACCTCCGTCGTTGCC<br>R: TAGTTGCACCTCACCCTC
OsPsbX1	Os07g0673550	Photosystem II protein X	F: GATGGTCCTCCCCGAC<br>R: CTGAGGAGGAAGTTCTTGAG
OsPsbX2	Os03g0343900		F: GATCTCCTCCTCTTCACAAG<br>R: ACAGAGACGCCGCAA
OsPsbY	Os08g0119800	Photosystem II protein Y	F: ATGCTTAAGCCGTCCAAGAT<br>R: CTCTTGAGCGAGATGCTAGG
OsPsbZ	OrsajCp010	Photosystem II protein Z	F: TGACTATTGCTTTCCAATTAGCTGT<br>R: TGACCAACCATCAGGAGAAGC
OsLhcb1.1	Os01g0720500	Chlorophyll a/b binding protein b	F: ACCATGGCACTCTCCTC<br>R: ACTTGCGCATCGTGAC
OsLhcb1.2	Os01g0600900		F: CATGGCCCTCTCGTCC<br>R: GACTTGCGCATGGTGAT
OsLhcb1.3	Os09g0346500		F: GACCGCGTCCTCTACCTCG<br>R: CCTTGAACCACACGGCCTC
OsLhcb1.4	Os09g0296800		F: TGGTGGTCAGGGTGTCATTG<br>R: CTAGAGCCTCAATCCCGCAG
OsLhcb2	Os03g0592500	Chlorophyll a-b binding protein[38]	F: AGACCACCAGCTTCCTC<br>R: ATGGTGATGCGGCCA
OsLhcb3	Os07g0562700	Type III chlorophyll a/b-binding protein	F: CTCCAATGCCCCCGT<br>R: CCCAGGTACTTCACCCTAT
OsLhcb4	Os07g0558400	Chlorophyll a/b-binding protein CP29	F: GTTCCTGGGGACGAGG<br>R: GCCGAACCCGAACCT
OsLhcb5	Os11g0242800	Minor chlorophyll a/b binding	F: AGACTACGGCTACGACCCTT<br>R: GCCGTTGATGATCCGGTAGT
OsLhcb6	Os04g0457000	Chlorophyll a/b-binding protein CP24	F: CTCGCCTCCACCTCC<br>R: CGACGGCATTGGCGA
2.3 CO_2 fixation			
OsRbcL	OrsaiCp23	RuBisco large subunit	F:AGGTGTTATACCGGTGGCTT<br>R:CTAAAGCCACCCGATTAGCT
OsRbcS1	Os02g0152400	Similar to ribulose 1,5-bisphosphate carboxylase	F: GTTCATCAACACGGCCA<br>R: CACCCTCCTCTGATCCC
OsRbcS2	Os12g0274700	small subunit	F: CCTTCCAGGGGCTCAA<br>R: GTTGCCGAAGCTGGAG
OsRbcS3	Os12g0291100		F: CCATTCCAAGGGCTCAAG<br>R: GACGTTGCCGAAGCC
OsRbcS4	Os12g0292400		F: TCGGTGATGGCTTCGT<br>R: ACGTTGCCAAAGCTCG

OsRbcS5	Os12g0291400		F: CCTCCGTTGCTCCCTT<br>R: CCGGCGCTGTTGGAG
OsPGK1	Os01g0800266	Phosphoglycerate kinase	F: AGATCCTTTCCCTGTCTCC<br>R: CATTGGCAGGAGTTGGG
OsPGK3	Os05g0496200		F: GCGAAGAAGAGCGTCG<br>R: TGATGTTCTGGTTGTCGTC
OsPGK5	Os10g0442100		F: ATCATTCAACATCATATCTGCAC<br>R: ACCTCTGCATTGAGTTTTTC
OsPGK6	Os03g0102400		F: TCCCACTCTCAGACTCAC<br>R: GACGAAGTCGTACTTGGAG
OsGAPD	Os03g0129300	Glyceraldehyde-3-phosphate dehydrogenase[39]	F: ATGGCCACACACGCA<br>R: CCTCAGTCCGGAGAAGTC
OsTPIM	Os09g0535000	Triosephosphate isomerase	F: CAAGTTCTTCGTTGGAGGC<br>R: CGTCTGCAAAAGTTGATTGAT
	Os03g0754200		F: GAGGAAGTTGCTCACTACTA<br>R: AAGATCAGGTTGTACTGCCA
OsTK1	Os06g0133800	Transketolase[40]	F: ATCCTCCAACATGACATTGC<br>R: GACTCCAAATCGGACATTGC
OsTK2	Os04g0266900		F: GCTGGGCCACGTCCTATTC<br>R: GTCGCGGTCGAACCAGTAG
OsPRK	Os02g0698000	Phosphoribulokinase	F: CGGGAAGAGCACCTTTATG<br>R: ATGAGTGTGTTTGAGTCTGG
OsTAL1	Os01g0926300	Transaldolase	F: TCTCTATGATCTCCAGGGTC<br>R: AATAAGGGGCAGCAAATCG
OsTAL2	Os08g0154300		F: TCACTGAGCTCGATGCC<br>R: GTCGGCGCAAACTTCT
OsCFR	Os03g0267300	Fructose-1,6-bisphosphatase[41]	F: GAGCGTGCGGTGCAT<br>R: GATCTCGTAGCTGCTCTTC
OsALDP	Os11g0171300	Fructose-bisphosphate aldolase	F: CAAGTCATCTTTCCTTCCCA<br>R: ACAACCATGGAGACAGTCA
OsPGI1	Os08g0232000	Glucose-6-phosphate isomerase	F: CATATGGTGTACATCCCTGG<br>R: GGAATATGCAGCTTCAAACA
OsPGI2	Os09g0465600		F: CAAGCTGTGGGAGAGGTA<br>R: GAGGAACTCCTCCGTGAA
3. $^1O_2$-mediated retrograde signaling pathway			
OsEX1	Os10g0485300	EXECUTER1 protein	F: GGGTTGATGGCGAGCTTTTG<br>R: CAGGAAGCTTCAACCGCCTA
OsBAP1	Os09g0526500	BON association protein 1	F: GAAGGAGACGCTGCGGGTGG<br>AGCTGAGGCAATGCAGGTGGC
OsNodL	Os06g0708700	Nodulin-like protein	F: ATTCCTCCTAGCCGCAATCG<br>R: AAGAACCTTGACGATGCCGT
OsOCT3	Os07g0571700	Organic cation/carnitine transporter	F: CTGTGCTTTCCTTGCACCAC<br>R: ATACTGCCAATCCGGCCAAA
OsAAA	Os06g0697500	AAA ATPase-like	F: AAAGTTGCGGGAGAGGAAGC

			R: GGTCGTAGATGTCGAACTCG
OsFTSH2	Os06g0669400	Cell division protease ftsH homolog	F: GTGTCGATGAAGCAAAGCAA<br>R: TCCGACAAACATCTCCACAA
OsFTSH6	Os06g0229066	ATP-dependent zinc metalloprotease	F: TCGACCTCCTCGTCAACTTC<br>R: TCCGGGAACTTCAAGAACTG
OsFTSH7	Os02g0649700		F: TGAACTGCGTGGACGTCTA<br>R: GTCTTAATCAGCGCATAGGT
OsFTSH9.1	Os01g0590700		F: TGGGAAATCACAAGAGGCCAT<br>R: TGGTGTATGGGATGAGCACC
OsFTSH9.2	Os01g0618800		F: AAGGTGCTGACAAGTTGGCT<br>R: CCACTTTCATGGTAGGCCGT
OsGSTU13	Os10g0530500	Glutathione-S-transferase Cla47	F: GTTCGAGGCGCTCCGCAG<br>R: CTTGGCCGCCTCGGCCTC
4. Genes involved in transcription of plastid genes			
OsSIG5	Os05g0586600	RNA polymerase sigma subunit E [42]	F: ACGAGGTCACCGACGACG<br>R: CTCTGCCGGATCACCA
OsSIG6	Os08g0242800	Similar to Sigma factor SIG6[42]	F: CACCTGACCTGAGTGTGGAC<br>R: CCCAAACATGTCCCCGATGA
OsRopTp	Os06g0652000	Nuclear-encoded plastid RNA polymerase [43]	F: TTCCGGTTGTTCAGCCCTAC<br>R: CAATTGCATCTCCCTCCCGT
OsrpoA	OrsajCp059	RNA polymerase alpha chain [44]	F: CGTTGGTATTGCCTTGCGAA<br>R: ACCGATTCTTCAATACCTGCT
OsrpoB	OrsajCp015	RNA polymerase beta chain [45]	F: TGCTAAGGAGAAGAAGAGGATTGA<br>R: CCGAAAATACCAGGTCCCCA
5. Internal reference gene for qPCRs			
OsActin	Os03g0718100	Actin gene	F: CTTCATAGGAATGGAAGCTGCGGGTA<br>R: CGACCACCTTGATCTTCATGCTGCTA

[a] The italic and bold italic indicated the nucleus- and chloroplast-encoded genes, respectively.

[b] Relevant literatures reported in rice were indicated.

References:

[1] Liu, W., Fu, Y., Hu, G., Si, H., Zhu, L., and Wu, C. (2007). Identification and fine mapping of a thermo-sensitive chlorophyll deficient mutant in rice (*Oryza sativa* L.). *Planta* 226(3), 785–795.

[2] Sun, C., Liu, L., Tang, J., Lin, A., Zhang, F., Fang, J. (2011). Rlin1, encoding a putative coproporphyrinogen iii oxidase, is involved in lesion initiation in rice. *J. Genet. Genomics* 38(1), 29–37.

[3] Wang, P., Li, C., Wang, Y., Huang, R., Sun, C., and Xu, Z. (2014). Identification of a geranylgeranyl reductase gene for chlorophyll synthesis in rice. *Springer Plus* 3(1), 1–9.

[4] Jung, S., Lee, H. J., Lee, Y., Kang, K., Kim, Y. S., and Grimm, B. (2008). Toxic tetrapyrrole accumulation in protoporphyrinogen ix oxidase-overexpressing transgenic rice plants. *Plant Mol. Biol.* 67(5), 535–46.

[5] Li, R. Q., Huang, J. Z., Zhao, H. J., Fu, H. W., Li, Y. F., Liu, G. Z., and Shu, Q. Y. (2014). A down-regulated epi-allele of the genomes uncoupled 4 gene generates a *xantha* marker trait in rice. *Theor. Appl. Genet.* 127(11), 491–501.

[6] Jung, K. H., Hur, J., Ryu, C. H., Choi, Y., Chung, Y. Y., and Miyao, A. (2003). Characterization of a rice chlorophyll-deficient mutant using the T-DNA gene-trap system. *Plant Cell Physiol.* 44(5), 463–72.

[7] Tian, X., Ling, Y., Fang, L., Du, P., Sang, X., and Zhao, F. (2013). Gene cloning and functional analysis of yellow green leaf 3 (ygl3) gene during the whole-plant growth stage in rice. *Genes & Genomics* 35(1), 87–93.

[8] Zhang, H., Li, J., Yoo, J. H., Yoo, S. C., Cho, S. H., Koh, H. J., et al. (2006). Rice chlorina-1 and chlorina-9 encode chld and chli subunits of mg-chelatase, a key enzyme for chlorophyll synthesis and chloroplast development. *Plant Mol. Biol.* 62(3), 325–37.

[9] Zhang, H., Liu, L., Cai, M., Zhu, S., Zhao, J., and Zheng, T. (2015). A point mutation of magnesium chelatase *OsChlI* gene dampens the interaction between ChlI and ChlD subunits in rice. *Plant Mol. Biol. Rep.* 33(6), 1975–1987.

[10] Kenji, F., Hiroshi, S., and Tadahiko, K. (2005). Identification of an active transposon in intact rice plants. *Mol. Genet. Genomics* 273(2), 150–157.

[11] Wang, P., Gao, J., Wan, C., Zhang, F., Xu, Z., Huang, X., et al. (2010). Divinyl chlorophyll(ide) a can be converted to monovinyl chlorophyll(ide) a by a divinyl reductase in rice. *Plant Physiol.* 153(3), 994–1003.

[12] Liu, H., Li, Q., Feng, Y., Zhu, F., Yi, S., and Tao, Y. (2016). Differential regulation of protochlorophyllide oxidoreductase abundances by virescent 5A (OsV5A) and virescent 5B (OsV5B) in rice seedlings. *Plant Cell Physiol.* 51, pcw151.

[13] Sakuraba, Y., Rahman, M. L., Cho, S., Kim, Y., Koh, H., and Yoo, S. (2013). The rice faded green leaf locus encodes protochlorophyllide oxidoreductase b and is essential for chlorophyll synthesis under high light conditions. *Plant J.* 74(1), 122–133.

[14] Kang, S. J., Fang, Y. X., Zou, G. X., Ruan, B. P., Zhao, J., and Dong, G. J. (2015). Gene encoding protochlorophyllide oxidoreductase b is involved in chlorophyll synthesis of rice. *Crop Sci.* 55(1), 284.

[15] Wu Z, Zhang X, He B, Diao L, Sheng S, Wang J (2007) A chlorophyll-deficient rice mutant with impaired chlorophyllide esterification in chlorophyll biosynthesis. *Plant Physiology* 145(1), 29–40.

[16] Morita, R., Kusaba, M., Yamaguchi, H., Amano, E., Miyao, A., & Hirochika, H. (2005). Characterization of chlorophyllide a oxygenase (cao) in rice. *Japanese Journal of Breeding* 55(3), 361–364.

[17] Lee, S., Kim, J. H., Yoo, E. S., Lee, C. H., Hirochika, H., and An, G. (2005). Different regulation of chlorophyll *a* oxygenase genes in rice. *Plant Mol. Biol.* 57(6), 805–818.

[18] Sato, Y., Morita, R., Katsuma, S., Nishimura, M., Tanaka, A., and Kusaba, M. (2008). Two short-chain dehydrogenase/reductases, non-yellow coloring 1 and nyc1-like, are required for chlorophyll b and light-harvesting complex ii degradation during senescence in rice. *Plant J.* 57(1), 120–131.

[19] Kusaba, M., and Tanaka, A. (2007). Rice non-yellow coloring1 is involved in light-harvesting complex ii and grana degradation during leaf senescence. *Plant Cell* 19(4), 1362–1375.

[20] Chen, H., Cheng, Z., Ma, X., Wu, H., Liu, Y., and Zhou, K. (2013). A knockdown mutation of yellow-green leaf2 blocks chlorophyll biosynthesis in rice. *Plant Cell Rep.* 32(12), 1855–1867.

[21] Chen, S. C. G., Cheng, M. C., Chung, K. R., Yu, N. J., and Chen, M. C. (1992). Expression of the rice chloroplast psaa-psab-rps14 gene cluster. *Plant Sci.* 81(1), 93–102.

[22] Chen, M. C., Cheng, M. C., and Chen, S. C. (1993). Characterization of the promoter of rice plastid PsaA-PsaB-rps14 operon and the DNA specific binding proteins. *Plant Cell Physiol.* 34(4), 577–84.

[23] Zhang, Y., and Yi, D. (2016). Molecular dynamics simulation and bioinformatics study on chloroplast stromal ridge complex from rice (*Oryza sativa* L.). *BMC Bioinformatics* 17(1), 1–12.

[24] Yadavalli, V., Neelam, S., Rao, A. S., Reddy, A. R., and Subramanyam, R. (2012). Differential degradation of photosystem I subunits under iron deficiency in rice. *J. Plant Physiol.* 169(8), 753–759.

[25] Mohanty, A., Grover, M., Chaudhury, A., Rizwan-Ul-Haq, Q., Sharma, A. K., Maheshwari, S. C., et al. (2000). Analysis of the activity of promoters from two photosynthesis-related genes *PsaF* and *peth* of spinach in a monocot plant, rice. *Indian Journal of Biochemistry & Biophysics* 37(37), 447–452.

[26] De, P. S., Hensgens, L. A., and Schilperoort, R. A. (1990). Structure and expression of a light-inducible shoot-specific rice gene. *Plant Mol. Biol.* 15(3), 399–406.

[27] Goh, C. H., Jang, S., Jung, S., Kim, H. S., Kang, H. G., and Park, Y. I. (2009). Rice phot1a mutation reduces plant growth by affecting photosynthetic responses to light during early seedling growth. *Plant Mol. Biol.* 69(5), 605–619.

[28] Higuchi-Takeuchi, M., and Matsui, M. (2011). Functional analysis of two isoforms of leaf-type

ferredoxin-NADP$^+$-oxidoreductase in rice using the heterologous expression system of *Arabidopsis*. *Plant Physiol. 157*(1), 96–108.

[29] Aoki, H., Doyama, N., Ida, S. (1994). Sequence of a cDNA encoding rice (*Oryza sativa* L.) leaf ferredoxin-NADP$^+$ reductase. *Plant Physiol.* 104(4), 1473–1474.

[30] Wang, G., Murase, J., Asakawa, S., and Kimura, M. (2009). Novel cyanophage photosynthetic gene *PsbA* in the floodwater of a japanese rice field. *FEMS Microbiol. Ecol.* 70(1), 79–86.

[31] Kapoor, S., Maheshwari, S. C., and Tyagi, A. K. (1993). Organ-specific expression of plastid-encoded genes in rice involves both quantitative and qualitative changes in mRNAs. *Plant Cell Physiol.* 34(6), 943–947.

[32] To, K. Y., Cheng, M. C., Suen, D. F., Mon, D. P., Chen, L. F. O., and Shu, C. (1996). Characterization of the light-responsive promoter of rice chloroplast PsbD-C operon and the sequence-specific DNA binding factor. *Plant Cell Physiol.* 37(5), 660–666.

[33] Gu, K., Luo, L., Su, C., and Zhai, H. (2001). Cloning and analysis of a cDNA encoding PsbL and PsbJ gene in rice chloroplast genome. *Acta Botanica Sinica, 43*(2), 210–212.

[34] Chen, S. C. G., Wu, S. P., Lo, P. K., Mon, D. P., and Chen, L. F. O. (1995). Regulation of plastid photosynthetic PsbK-I-D-C, gene expression by light in rice plants. *Physiologia Plantarum* 93(4), 617–623.

[35] Li, T., Gong, C., and Wang, T. (2010). The rice light-regulated gene ra68 encodes a novel protein interacting with oxygen-evolving complex PsbO mature protein. *Plant Mol. Biol. Rep.* 28(1), 136–143.

[36] Wang G, Murase J, Susumu A, Makoto K (2009). Novel cyanophage photosynthetic gene psba in the floodwater of a japanese rice field. *FEMS Microbiology Ecology, 70*(1), 79-86.

[37] Ishida, S., Morita, K. I., Kishine, M., Takabayashi, A., Murakami, R., and Takeda, S. (2011). Allocation of absorbed light energy in PSII to thermal dissipations in the presence or absence of PsbS subunits of rice. *Plant Cell Physiol.* 52(10), 1822–1831.

[38] Kang, K., Kim, Y. S., Park, S., and Back, K. (2009). Evaluation of light-harvesting complex proteins as senescence-related protein markers in detached rice leaves. *Photosynthetica* 47(4), 638–640.

[39] Zhang, X. H., Rao, X. L., Shi, H. T., Li, R. J., & Lu, Y. T. (2011). Overexpression of a cytosolic glyceraldehyde-3-phosphate dehydrogenase gene *Osgapc3*, confers salt tolerance in rice. *Plant Cell Tiss. Organ Cult.* 107(1), 1–11.

[40] Suzuki, Y., Kondo, E., and Makino, A. (2017). Effects of co-overexpression of the genes of rubisco and transketolase on photosynthesis in rice. *Photosyn. Res.* 131, 281–289.

[41] Lee, S. K., Jeon, J. S., Börnke, F., Voll, L., Cho, J. I., and Goh, C. H. (2008). Loss of cytosolic fructose-1,6-bisphosphatase limits photosynthetic sucrose synthesis and causes severe growth retardations in rice (*Oryza sativa* L.). *Plant Cell Envir.* 31(12), 1851–1863.

[42] Kubota, Y., Miyao, A., Hirochika, H., Tozawa, Y., Yasuda, H., Tsunoyama, Y., et al. (2007). Two novel nuclear genes, *OsSig5* and *OsSig6*, encoding potential plastid sigma factors of RNA polymerase in rice: tissue-specific and light-responsive gene expression. *Plant Cell Physiol.* 48(1), 186–192.

[43] Kusumi, K., Yara, A., Mitsui, N., Tozawa, Y., & Iba, K. (2004). Characterization of a rice nuclear-encoded plastid RNA polymerase gene OsRpoTp. *Plant Cell Physiol.* 45(9), 1194–1201.

[44] Sun, C. R., and Sugiura M. (1989). Cloning and sequencing of *Rpoa* gene from rice chloroplast genome. *Acta. Botanica Sinica*, 31: 763–769.

[45] Shimada, H., Fukuta, M., Ishikawa, M., and Sugiura, M. (1990). Rice chloroplast RNA polymerase genes: the absence of an intron in *Rpoc1* and the presence of an extra sequence in *Rpoc2*. *Molecular Genetics and Genomics* 221(3), 395–402.
