# Supplementary material for: The xantha Marker Trait Is Associated with Altered Tetrapyrrole Biosynthesis and Deregulated Transcription of PhANGs in Rice
Source: Front Plant Sci. 2017 May 31;8:901. doi: 10.3389/fpls.2017.00901 (PMC5449477; doi:10.3389/fpls.2017.00901)
Supplement: Supplementary file 2 [file Image_1.PDF]

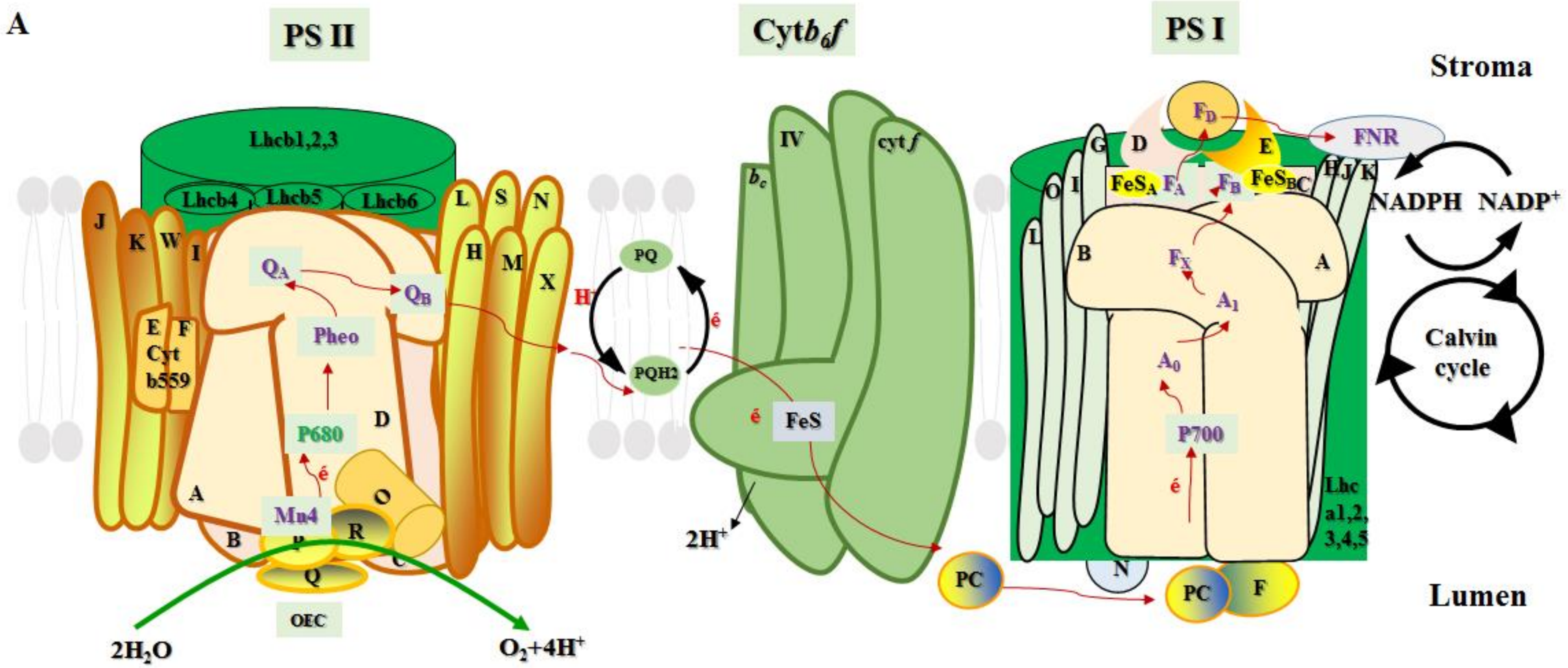
A
PS II
Cytb6f
PS I
Stroma
Lhcb1,2,3
Lhcb4
Lhcb5
Lhcb6
J
K
W
I
L
S
N
H
M
X
E
F
Cyt
b559
QA
QB
Pheo
P680
D
A
Mn4
B
P
R
O
C
Q
OEC
2H2O
O2+4H+
H+
PQ
PQH2
é
IV
cyt f
bc
FeS
2H+
FD
FNR
G
D
E
FeSA
FA
FB
FeSBC
HJ
K
O
I
L
B
FX
A1
A0
P700
A
NADPH
NADP+
Calvin
cycle
Lhc
a1,2,
3,4,5
N
PC
F
Lumen

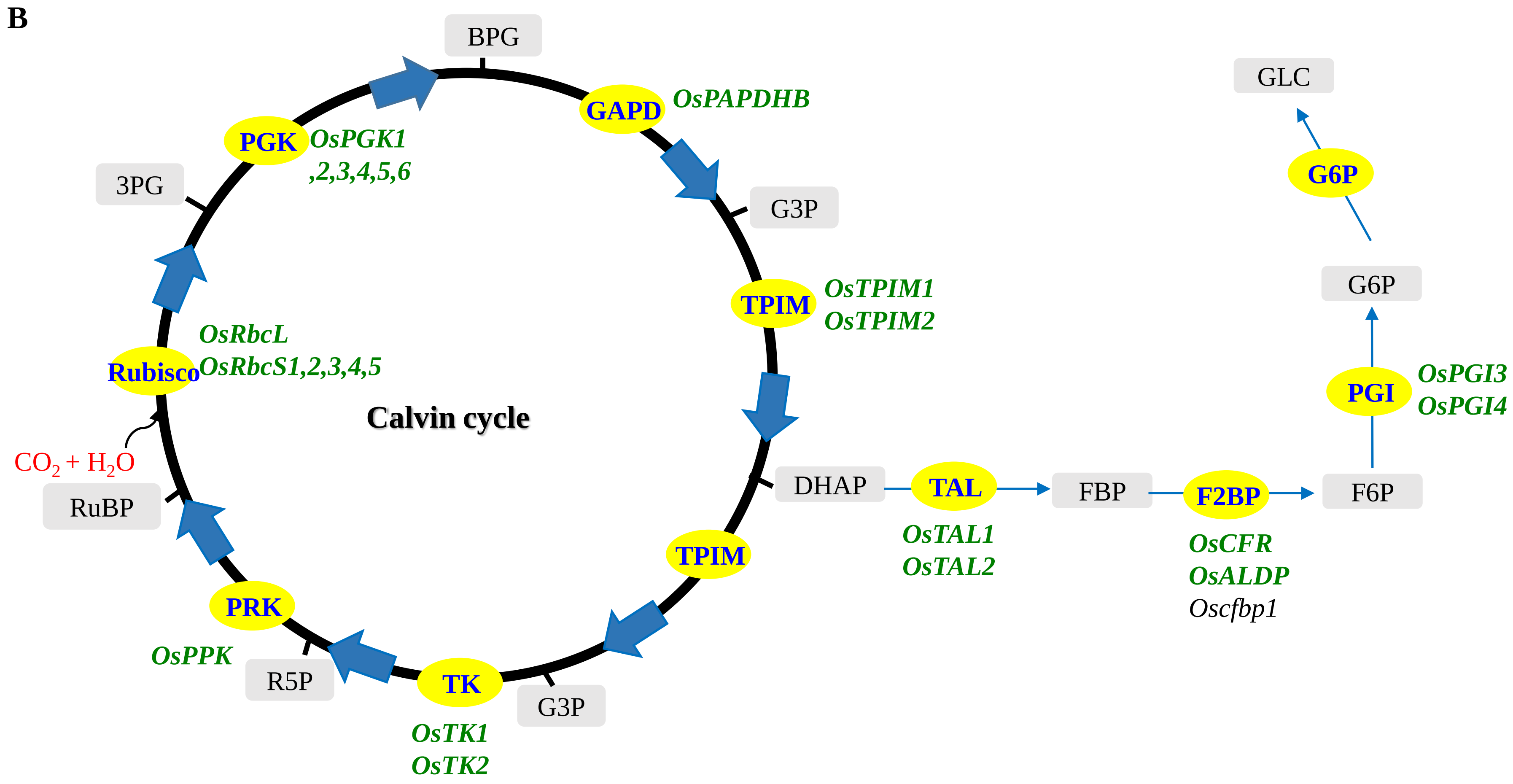
B
BPG
GAPD
OsPAPDHB
G3P
TPIM
OsTPIM1
OsTPIM2
DHAP
TAL
OsTAL1
OsTAL2
FBP
F2BP
OsCFR
OsALDP
Oscfbp1
F6P
PGI
OsPGI3
OsPGI4
G6P
G6P
GLC
TPIM
G3P
TK
OsTK1
OsTK2
R5P
PRK
OsPPK
RuBP
$CO_2 + H_2O$
Rubisco
OsRbcL
OsRbcS1,2,3,4,5
3PG
PGK
OsPGK1
,2,3,4,5,6
Calvin cycle

FIGURE S1 | Schematic models of photosynthetic progress in rice. (A) Schematic figures of the light reaction in photosynthesis. Rice photosynthetic reaction depend on two photosystems of PS I (Photosystem I) and PS II (Photosystem II) and the cyt (cytochrome) b_6f complex. The subunits of PS I composition include PsaA—PsaL, light harvesting chlorophyll binding protein Lhca1—Lhca4, and also two subunits of Psa N and Psa O. PSII subunit componets are the major intrinsic proteins of PsbA (D1), PsbB (CP47), PsbC(CP43) and PsbD (D2), the polypeptide of PsaE and PsbF, cyt b_{559}, and O_2 evolution activity (OEC) unit (PsaO, PsaP and PsbQ). PsbS, the unique subunit of eukaryotes, and other polypeptide (PsbH-N, PsbR, PsbT, PsbW-PsbZ) are also indicated. The electron transport chain (ETC) of rice chloroplast are shown in red arrows. The components of ETC are indicated as purple fonts, incuding $(Mn)_4$, P680, Phe, Q_A, Q_B of PS II, PQH2 and PC of Cyt b_6f, and P700, A_0, A1, Fx, F_A, F_B, F_d and FNR in PS I. CP: chlorophyll-binding protein; PC: plastocyanin; F_D: ferredoxin; FNR: ferredoxin NADP oxidoreductase; Cyt: cytochrome; P680: primary eletron donor of PS II; Pheo: pheophytin a; QA, QB: quinone electron acceptors; F_X: 4Fe-4S; P700: primary electron donor of PS I; FA, FB: Fe-S electron acceptors; A_0: chlorophyll a; A_1: phylloquinone. This figure was modified from the review of Najafpour and Allakhverdiev (2011). (B) Schematic figures of the black reaction in photosynthesis. The concrete steps of CO_2 fixation in rice are indicated. The enzymes and enzemic products are shown in blue bold folds with yellow backdrop and black folds with gray backdrop, respectively. The rice encoding genes for CO_2 fixation are shown in green bold folds, while the rice mutants now have been reported are black italic.Rubisco: Ribulose bisphosphate carboxylase oxygenase; PGK: Phosphoglycerate kinase; GAPD: Glyceraldehyde—3-phosphate dehydrogenase; TPM: Triose-phosphateisomerase; TAL:transaldolase; F2BP: Fructose-1,6-bisphosphatase; PGI: Glucose-6-phosphate isomerase; G6P:Glucose-6-phosphatase; PRK: Phosphoribulokinase; TK: Transketolase; RuBP: Ribulose -1,5-bisphosphate; 3PG: 3-phosphoglycerate; BPG: 1,3-phosphoglycerate; G3P: Glyceraldehyde 3-phosphate; DHAP: Dihydroxyacetone phosphate; R5P: Ribulose 5-phosphate.

A B

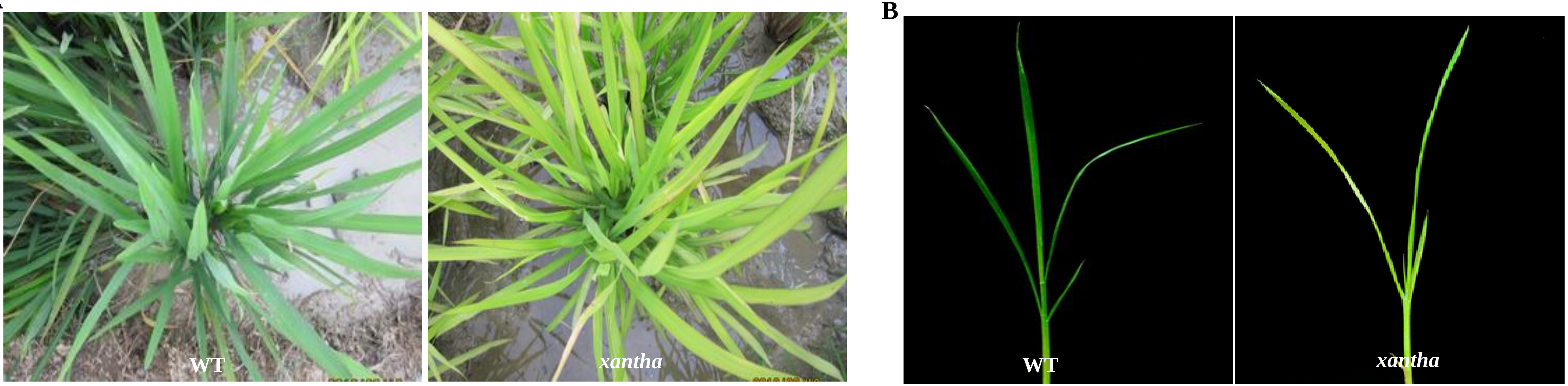

FIGURE S2 | The leaf color phenotypes of Longtefu B (WT) and its *xantha* mutant Huangyu B. (A) Plants at tillering stage in a paddy field. (B) Phenotypes of 35-days-old seedlings in a growth chamber under a 16-h light (1,000 μmol photons m^{-2} s^{-1}; 30 °C):8-h dark (26 °C) photoperiod.

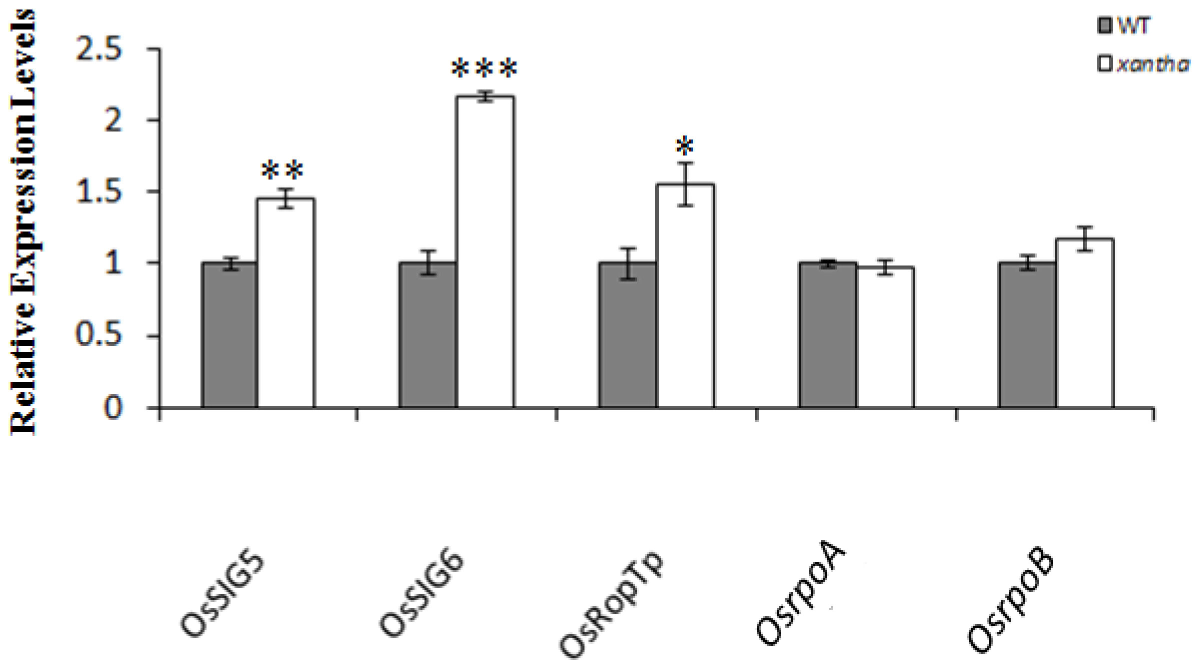

FIGURE S3 | Expression of genes involved in the transcription of plastid genes. The relative transcript levels in *xantha* plants (empty bar) were reported relative to their respective genes in wild-type (gray bar) after normalizing to internal control *OsActin*. Error bars represent the standard error. *, **, and *** represent significant differences between wild-type and *xantha* mutant values at $P < 0.05$, $P < 0.01$, and $P < 0.001$, respectively.
